# Investigation into the Microstructure and Hardness of Additively Manufactured (3D-Printed) Inconel 718 Alloy

**DOI:** 10.3390/ma16062383

**Published:** 2023-03-16

**Authors:** Abdulaziz Kurdi, Abdelhakim Aldoshan, Fahad Alshabouna, Abdulaziz Alodadi, Ahmed Degnah, Husain Alnaser, Thamer Tabbakh, Animesh Kumar Basak

**Affiliations:** 1 The Center of Excellence for Advanced Materials and Manufacturing, King Abdulaziz City for Science and Technology, P.O. Box 6086, Riyadh 11442, Saudi Arabia; 2 Advanced Manufacturing Technology Institute, King Abdulaziz City for Science and Technology, P.O. Box 6086, Riyadh 11442, Saudi Arabia; 3 Advanced Materials Technology Institute, King Abdulaziz City for Science and Technology, P.O. Box 6086, Riyadh 11442, Saudi Arabia; 4 Material Science and Engineering Department, University of Utah, 135 S 1460 E, WBB 112, Salt Lake City, UT 84112, USA; 5 Microelectronics and Semiconductors Institute, King Abdulaziz City for Science and Technology, P.O. Box 6086, Riyadh 11442, Saudi Arabia; 6 Adelaide Microscopy, The University of Adelaide, Adelaide, SA 5005, Australia

**Keywords:** powder laser bed fusion, microstructure, Inconel 718, hardness

## Abstract

Additive manufacturing (AM) of Ni-based super alloys is more challenging, compared to the production other metallic alloys. This is due to their high melting point and excellent high temperature resistance. In the present work, an Inconel 718 alloy was fabricated by a powder laser bed fusion (P-LBF) process and investigated to assess its microstructural evolution, together with mechanical properties. Additionally, the alloy was compared against the cast (and forged) alloy of similar composition. The microstructure of the P-LBF-processed alloy shows hierarchy microstructure that consists of cellular sub-structure (~100–600 nm), together with melt pool and grain boundaries, in contrast of the twin infested larger grain microstructure of the cast alloy. However, the effect of such unique microstructure on mechanical properties of the L-PBF alloy was overwritten, due to the absence of precipitates. The hardness of the L-PBF-processed alloy (330–349 MPa) was lower than that of cast alloy (408 MPa). The similar trend was also observed in other mechanical properties, such as Young’s modulus, resistance to plasticity and shear stress.

## 1. Introduction

The use of additive manufacturing (AM) of metallic alloys is on rise as the fabrication process is efficient and offers the near-net shape fabrication of components. In this process, two-dimensional (2D) layers of materials are stacked-up on the top of each other, which is followed by the solidification of the layers by heat input [1,2]. The process is controlled by computer and provide the input parameters, such as slicing geometry, shape, layer thickness, and other required information from computer-aided design (CAD) data sets. The systems are classified according to the method of heat input used to consolidate the materials. For example, if the electron beam is utilized as the heat source, then the method is known as electron (E)-beam AM; if laser beam is in use, then the method is termed as laser beam AM. Out of diverse AM systems, the laser powder bed fusion (L-PBF) is a popular process [3,4], particularly for AM of metallic materials [5]. In this technique, a thin layer (~30–200 µm) of powders of desired material is spread out on a base plate inside a chamber, after which it is rastered with a laser beam to solidify the power. Once solidified, the next layer of powder is applied at the required thickness, and the procedure is continued until the anticipated shape has been fabricated. Under non-equilibrium conditions, for example, during L-PBF processing of metallic alloys, the cooling rate is very high (103–108 K/s), which works together with intense heat exposure for a fraction of a second [6,7]. These conditions impose excessive superheating and undercooling in the melt, which accelerates the nucleation rate. As a result, metastable microstructures that form at high temperatures are retained at room temperature with suppressed gains growth [8]. Therefore, the microstructure of the L-PBF-processed metallic alloys is unique and significantly different than that of cast alloys of a similar composition. However, development of these unique microstructure does not come unconditionally, as will be explored in the current research.

Inconel 718 is a nickel-based superalloy which is a solid solution-strengthened/precipitation-hardened product. It possess high resistance against oxidation and corrosion, high strength, even at elevated temperatures and is thus widely used in gas turbine and chemical processing industries [9]. Traditionally, components made from this alloy are crafted via casting followed by machining, which is challenging due to the high hardness of Inconel. Therefore, the use of AM opens up a new era for the processing of such alloy in near net-shape structure. As high heat input is required to consolidate the powders of Inconel alloy, hence L-PBF is used in a preliminary manner to fabricate components of this alloy. However, a major challenge to that is the generation of cracking [10,11,12], similar to what occurs during welding [13], due to the presence of high solidification rates. Different methods were proposed to mitigate this problem, including the introduction of changes to processing parameters and the composition of the material system. Tomus et al. [10] reported that, by lowering the Mn and Si element content in the starting powder, the hot cracking behavior can be reduced significantly by suppressing micro-segregation at grain boundaries. However, Harrison et al. [12] found that this could not be the case and placed emphasis on ultimate strength and thermal shock resistance of the material system. This was gained by increasing the amount of solid solution strengthening elements (e.g., Co, W and Mo), and at the same time, by reducing the Mn and C content. The outcome was about a 57% reduction in crack density. On the other note, input parameters, such as laser power and scan-speed can also be adjected towards that. However, care should be taken, as high power can favor coarser grains in the microstructure and introduce crystallographic texture. Conversely, a lower laser power may introduce unconsolidated regions and porosities [14]. According to the literature survey, it can be observed that most of the reports on L-PBF Inconel alloy are on process parameter optimization to achieve dense components in the absence of hot cracking, with few reports on their mechanical properties, mainly on tensile strength [9,10]. However, hardness of any given material is of prime importance, which could be correlated to the plastic and elastic flow behavior of the material [15]. In view of that, the novelty of the present work is the in-depth investigation on the micro- and nanohardness of L-PBF-processed Inconel 718 alloy in terms of the plastic and elastic flows of the materials.

Hence, the objective of the current study is to investigate the microstructural and micro-mechanical properties of the Inconel 718 alloy, fabricated via L-PBF process. Towards that end, cast Inconel 718 alloy of similar composition was used as a benchmark material.

## 2. Experimental

The starting material used in this work was Inconel 718 powder procured from Selective Laser Melting (SLM solutions, Lübeck, Germany). This powder was gas-atomized, with a spherical shape in macrostructure and a particle size ranging from 10–45 μm. The most important parameters towards L-PBF are laser power, scan speed, spot size, hatch spacing, and layer thickness [16,17]. The heat input by the laser power, i.e., energy density (ED), can be expressed as [18,19]:

(1) $$ED=\frac{P}{v_{s}ht}\;\;$$

where *P* resemble laser power, vs. is scanning speed, *h* is hatch distance, and *t* represents layer thickness. According to the literature, the energy densities for Inconel powder consolidation fall in the range of 20–160 J/mm^3^ [9,20]. Based on this information, to maintain an energy density of about 70 J/mm^3^, the following parameters were employed in the present study: 280 W of laser power, 0.1 mm of hatch distance, 0.1 mm of layer thickness and 800 mm/min of scan speed. The equipment used was a SLM 250 HL printer (SLM Solutions Group AG) which comes with a 400 W Nd:YAG laser. To keep the oxidation of the powder at a lower level, the oxygen content was maintained below 0.5 wt. % during the printing process in a closed system by purging argon gas. To start a solid build up, the base plate was heated at 100 °C, and the scan direction was rotated by 45° between successive layers to mitigate stress build up. The final samples were given in the form of a 10 mm cube. After fabrication, post-stress relief was conducted at 220 °C to relieve the build-up residual stress that took place in course of fabrication [21].

The cast Inconel 718 alloy was commercially procured from Rolled Alloys Ltd. (Singapore) in the form of a round bar, following a nominal composition (wt. %) as provided by the supplier: 52.5 Ni-19 Cr-3.0 Mo-5.1 Nb-0.90 Ti-0.50 Al-18 Fe. According to the supplier, the alloy was first melted in a vacuum induction furnace (968 °C). Subsequently, the allow was subjected to forging to give the bar shape and then subjected to solution seat treatment to precipitation-harden the substance [22]. Hence, it is termed as ‘cast alloy’ in the rest of the manuscript to distinguish it from the L-PBF alloy. This cast alloy was used as a benchmark material with which to compare the properties of the currently investigated Inconel 718 L-PBF alloy.

The density of the sample was measured according to the Archimedes principle. The sample was cut with the help of slow-speed diamond saw, and then mounted in resin, which was followed by metallographic polishing. The final polishing was conducted in colloidal silica to obtain a scratch-free polished surface. The microstructural characterization of the samples was conducted in a field emission scanning electron microscope (FESEM, Hitachi SU 7000), equipped with elemental X-ray dispersive (EDS) and electron back scattered diffraction (EBSD) detectors. The microhardness was measured on the surface of the polished samples by using a Clark microhardness (CM-100AT) tester at 100 g, 300 g and 500 g loads, and at a 10 s indentation time. A total of 15 indentations were conducted on each sample, and the average, with standard deviation, was reported and used for analysis purposes. Nanoindentaiton was carried out by an IBIS nanoindentor (Fischer-Cripps Lab., Sydney, Australia). A Berkovich tip was used for nanohardness measurement, with a peak load of 100 mN and a holding time of 3 s at the peak load. A minimum of 50 indentations were carried out in each case, and the average value (with standard deviation) was reported and used for analysis purpose.

## 3. Results and Discussion

**Figure 1:**
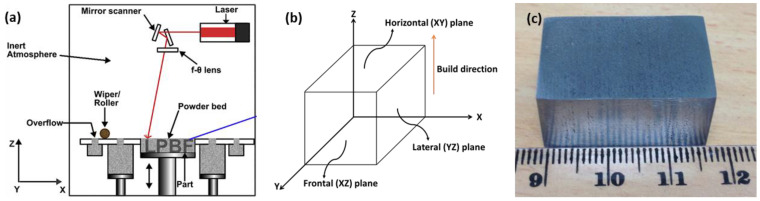
Schematic of (**a**) L-PBF process [25] and (**b**) sample orientation and associated investigated planes, together with (**c**) optical photographs of the L-PBF-processed sample.

AM components are well known for their directionality in microstructure and in mechanical properties in relation to their build orientation [23,24]. Therefore, to obtain a better understanding on this, we conducted microstructural and mechanical characterization of the presently investigated samples on three induvial planes in relation to the build direction, as shown schematically in Figure 1.

The plane perpendicular to the build direction is referred as the horizontal (XY) plane and the planes parallel to the build direction are referred as frontal (XZ) and lateral (YZ) planes. The theoretical density (TD) of Inconel 718 is 8.19 g/cc [17]. With respect to that, density of the presently investigated L-PBF-fabricated Inconel 718 was 98.6%, as recorded in Table 1, whereas the density of cast alloy was 99.8% of theoretical density.

### 3.1. Microstructure of the Alloys

#### 3.1.1. Microstructural Investigation by Electron Microscopy

**Figure 2:**
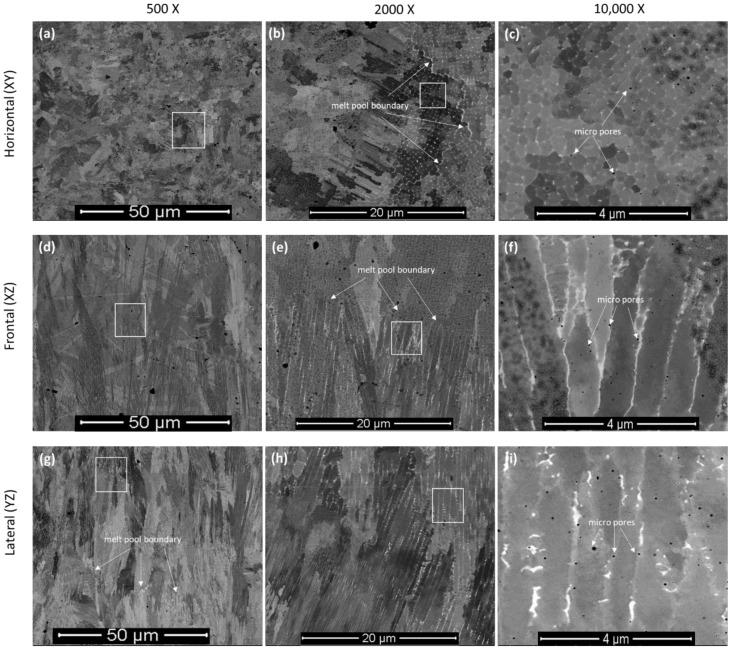
SEM micrographs of the P-LBF-processed Inconel 718 after metallographic polishing, at different magnification, on: (**a**–**c**) horizontal, (**d**–**f**) frontal and (**g**–**i**) lateral in relation to the build direction. The white boxes indicate the areas from where the higher magnification images were obtained.

Backscattered secondary electron (BSE) images on different planes of L-PBF-processed Inconel 718, with respect to the build orientation, after metallographic polishing is shown in Figure 2 at different magnifications (×500, ×2000 and ×10,000) to unravel the microstructure at different length scales. In general, the microstructure contains some metallurgical pores, as indicated by arrows, which arise due to gas entrapment during the fabrication process [26,27]. These pores are spherical in shape and <1 µm in diameter. As marked in Figure 2, the pores distributed all over the microstructure without any preferential arrangement. Like the microstructure of the other L-PBF-processed metallic alloys system, the Inconel 718 also exhibits melt-pool predominant microstructure [28,29]. The melt pools correspond to the laser tracks that are several microns (~100 µm) in width. As can be seen from Figure 2, the microstructure of the alloy can be categorized in two levels: (i) the grain and laser track boundaries, as evident in relatively lower magnification, and (ii) the cell boundaries at relatively higher levels of magnification. For instance, the microstructure at 500- and 2000-times magnification shows the existence of melt-pool boundaries. In horizontal planes (Figure 2a,b), this appears as a zig-zag pattern, whereas in frontal (Figure 2d,e) and lateral planes (Figure 2g,h), it appears as a hemispherical shape with a typical diameter of around 80–120 μm. The individual grains can be distinguished by the changes in the contrast within the melt pools, as observed in 10,000 times-magnified images (Figure 2c,f,i). Along build directions (both frontal and lateral planes), the grains show typical elongated morphology (epitaxial growth) through the layers. In addition, typical cellular/dendritic solidification microstructures (Figure 2c,f,i) are evident, being around 100–600 nm in width and several microns in length. These are in line with previous reports on the same material system [10,12,30]. The precipitates tends to preferentially reside along the cell boundaries (Figure 2c,f,i) and thus form the cellular structure [31]. The thickness of the cell boundaries is in the range of about 50 nm (Figure 2c,f,i). This unique development in microstructure was attributed to the fast solidification of the alloy during the P-LBF process. Under such circumstances, the solubility of the alloying elements in the melt is higher and they begin to solidify in a cellular morphology with dendritic characteristics. According to Li et al. [8], the melt-pool center may experience a temperature of about 1712.15 K. This high heat input caused superheating in the melt. However, the interaction time and volume of melt is minimum, and facilitates inhomogeneous microstructural formation (at micro-/nano-scale) in the melt pools. The high cooling rate retains such microstructural formation and cellular growth into room temperature [8].

**Figure 3:**
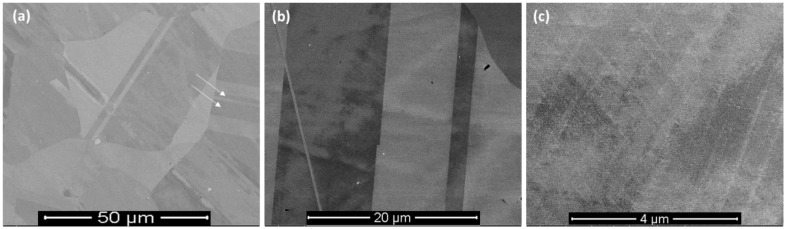
SEM micrographs of cast Inconel 718 after metallographic polishing at different magnification: (**a**) ×500, (**b**) ×2000 and (**c**) ×10,000. The white arrows indicate the location of the twins.

In contrast to that, cast alloy of similar composition shows large-grained microstructures (Figure 3) with the presence of numerical twins (marked with arrows in Figure 3a), which are typical characteristics of such Inconel 718 alloy. During casting process, the solidification rate is much slower, as is undercooling, compared to that of the L-PBF process [32] that favored large-grained microstructure. Further investigations to investigate the gain size and grain size distribution were conducted with the help of EBSD, as reported in the next section.

#### 3.1.2. Grain Orientation and Texture of the Alloys

**Figure 4:**
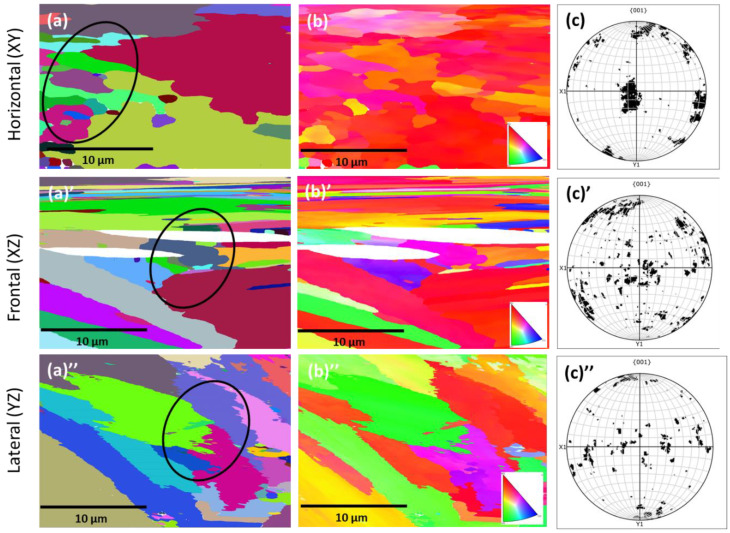
Grain boundary (GB) (**a**,**a’**,**a”**), inverse pole figure (IPF) (**b**,**b’**,**b”**) and pole figure (PF) (**c**,**c’**,**c”**) maps on the L-PBF-processed Inconel 718 alloy: (**a**–**c**) horizontal, (**a’**,**b’**,**c’**) frontal and (**a”**–**c”**) planes in relation to the build direction (BD). The black circles indicate the area where changes in gain orientation took place.

The outcome of the EBSD investigation is shown in Figure 4, which exhibits the crystallographic orientation with respect to build direction. Figure 4a,a’,a” show the grain boundary (GB) map, Figure 4b,b’,b” show the inverse pole figure (IPF) map and Figure 4c,c’,c” show the pole figure (PF) map. The inverse pole figures in frontal and lateral planes represent the texture, parallel to the build direction and as evident, majority of the grains incline to grow parallel to the build direction. There was also some small amount of grains that grew below 30–45° angles with respect to the Z axis. Regarding the crystallographic orientation, texture along the 〈100〉 crystal direction, parallel to the build direction, was noticed. From the GB maps (Figure 4a,a’,a”), the distinction of core and melt-pool boundaries (as marked with ovals) are evident. As mentioned in Section 3.1.1, the core contains relatively elongated grains, whereas the melt-pool boundaries have equiaxed grains due to the exposure of multiple melting cycles. As the scan trajectory was rotated 45° to the fabrication process in order to avoid stress build-up, no obvious texture was noticed in the pole figure maps (Figure 4c,c’,c”).

**Figure 5:**
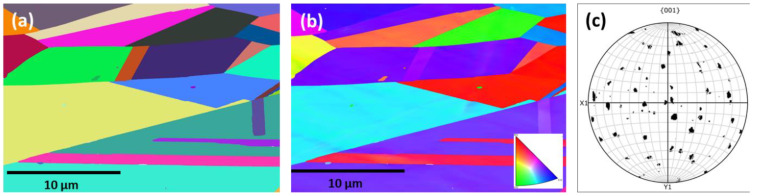
EBSD maps on cast Inconel 718 alloy: (**a**) Grain boundary (GB), (**b**) inverse pole figure (IPF), and (**c**) pole figure (PF) maps.

In contrast to the ESBD maps on the P-LBF-processed alloy, EBSD maps of the cast alloy exhibit a striking difference, as shown in Figure 5, where the relatively larger gains are quite evident in the presence of twins.

**Figure 6:**
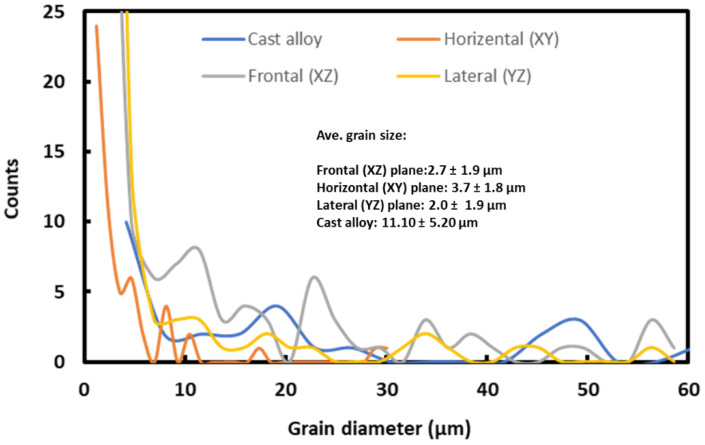
Grain size distribution, with mean grain size of L-PBF-processed and cast Inconel 718 alloy, as determined from EBSD data analysis.

In addition to the texture evaluation, grain size distribution was also obtained from the EBSD investigation, as shown in Figure 6. From Figure 6, it was clear that the average grain size of L-PBF-processed alloy was about 1.4–1.7 μm, which was a couple of a magnitude larger than the cell size (Figure 4b’). In contrast, the grain size of the cast alloy was about 3.4 ± 0.2 µm.

### 3.2. Mechanical Properties of the Alloys

#### 3.2.1. Vickers’s Hardness

**Figure 7:**
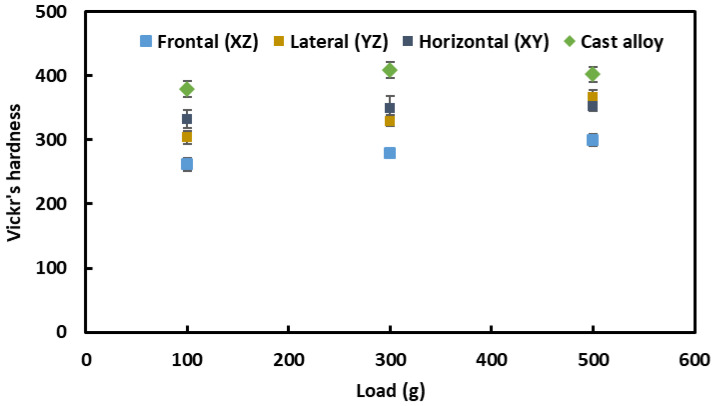
Vickers’s hardness of L-PBF and cast Inconel 718 alloy.

The Vickers’s hardness of the materials was investigated under three different loads: namely, 100 g, 300 g and 500 g, and the evolution of hardness is shown in Figure 7. There is a slight increase in hardness with the increase in indentation loads, with similar trends for all the samples. This slight increase in hardness may be associated with local inhomogeneity under the indentation imprint. As can be seen from Figure 6, the hardness of cast alloy is somewhat higher (408 HV_0.3_) than that of the L-PBF-prepared samples (330–349 HV_0.3_), as reported in Table 1. In the literature, similar hardness values were reported for the as-built L-PBF Inconel 718 alloy, i.e., 296 HV [33]–320 HV [34]. The reported hardness of the cast alloy in the literature was 469 HV [33], higher than that found in the current investigation. Therefore, in general, the hardness of L-PBF-processed Inconel is lower than that of cast alloy. The origin of this lies in the microstructural evolution and the distribution of laves, carbide phases, and γ” precipitation, as reported by Zhou et al. [33]. As the presently investigated L-PBF Inconel 718 was not heat-treated/aged, the benefits of solution and precipitation hardening of this alloy were overwritten. Therefore, it is recommended that researchers to carry out appropriate heat treatment/aging of such alloys processed by L-PBF prior to service applications.

**Figure 8:**
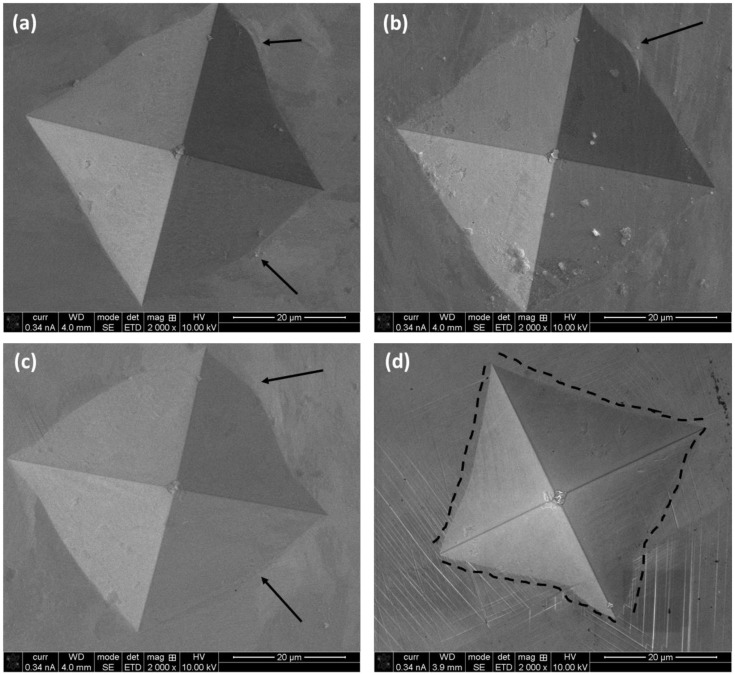
SEM images on residual imprints after Vickers’s indentation on L-PBF Inconel 718 alloy: (**a**) horizontal (XY), (**b**) frontal (XZ), (**c**) lateral (YZ) planes with respect to the build direction, and (**d**) cast alloy. The black arrows indicate the ‘pile-up’ and the dotted line indicate the profile of the indentation mark.

The SEM images of the residual imprints of hardness are shown in Figure 8. It is evident that there are no cracks along the corners of L-PBF-prepared samples (Figure 8a–c). However, pile-up does occur, as indicated by the arrows. The pile-up is generally associated with the ease of plastic flow of the materials [35]. In contrast, the cast alloy shows the shrinkage of the edges, which is similar to the one reported in the literature [36] and related to the high hardness of the material, i.e., higher resistance against the flow of the material. This was explored further by the nanoindentation, as reported in the next section.

#### 3.2.2. Plastic and Elastic Behavior under Loading

**Figure 9:**
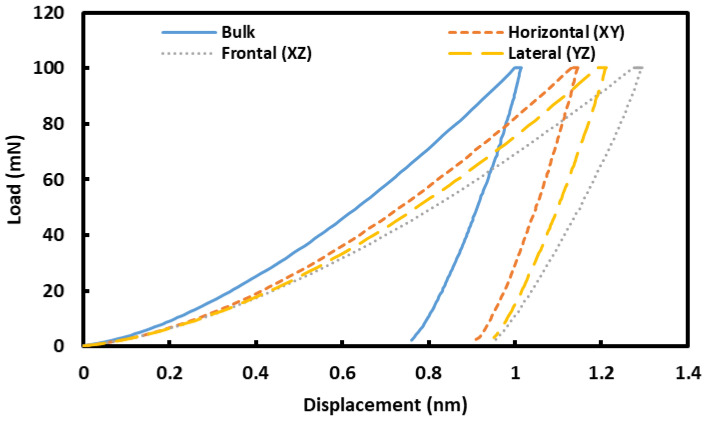
Representative load–displacement graphs on different plans of L-PBF and cast Inconel 718 alloy.

Representative load–displacement graphs, obtained during nanoindentation on the presently investigated samples, are shown in Figure 9. Figure 9 contains a single load–displacement graph for each sample, allowing for better visualization and comparison. As reported in the experimental section, 50 nanoindentation measurements were carried out in each sample and the outcome is presented in the Supplementary Material section (Figures S1–S4). As evident from Figure 9, all the samples (both L-PBF and cast) exhibit elastic–plastic behavior and the plasticity of cast sample is lower than that of L-PBF samples (lower residual displacement). This was not a surprise, in view of the Vickers’s hardness data and residual indentation imprint, as reported in Section 3.2.1. Among different planes of L-PBF alloy, the curves are quite similar and there is similar residual displacement.

**Figure 10:**
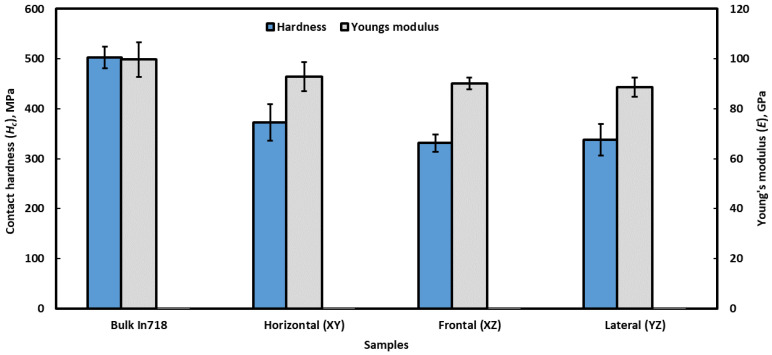
The nanomechanical properties of L-PBF and cast Inconel 718 alloy: Contact hardness (*H_c_*) and Young’s moduli (*E*).

A number of mechanical properties of the investigated alloys, such as contact hardness (*H_c_*) and Young’s modulus (*E*), were obtained by analyzing the unloading portion of the load–displacement curves [37], as shown in Figure 10 and also reported in Table 1. Both the hardness and Young’s modulus of the L-PBF-processed alloy are somewhat lower than those of cast alloy. Among different planes of the L-PBF-processed alloy, the horizontal plane exhibits a marginally higher properties than the frontal and lateral planes. This can be attributed to the microstructural evaluation of the L-PBF-processed alloy. In accordance with the microstructural arrangement, as reported in Section 3.1, during nanoindentation, melt-pool boundaries are parallel to the loading direction in the frontal and lateral planes. However, in the case of horizontal planes, such boundaries are perpendicular to the loading direction. Hence, there is a higher chance that the indentation resides in the melt-pool boundaries in the case of frontal and lateral planes and that it thus exhibits relatively lower mechanical properties. This in turn gives rise to anisotropy of mechanical properties of the alloy.

Further to that, the elastic–plastic deformation behavior of the alloys was analyzed according to the Sakai model [38]. According to this theory, the total displacement (*h_t_*) caused by any indentation can be divided into two segments: elastic (*h_e_*) displacement and plastic (*h_p_*) displacement. Thus, the total displacement (*h_t_*) can be summarized as follows [38]:

(2) $$h_{t}=h_{e}+h_{p}\;\;$$

Equation (1) can be written as [38,39]

(3) $$h_{t}=\sqrt{\frac{P_{max}}{\alpha_{2}E^{\prime }}}+\sqrt{\frac{P_{max}}{\alpha_{1}H_{T}}}$$

where *P_max_* is the maximum peak load, *α*_2_ is constant (4.4 for Berkovich indenter), $E^{\prime }=E/\left(1-v^{2}\right)$ (plastic strain), *α*_1_ is a non-dimensional constant (24.5 for Berkovich indenter), *v* is Poisson’s ratio, and *H_T_* is the resistance to plasticity. By re-arranging the equations, the resistance to plasticity *H_T_* can be expressed as [40]:

(4) $$H_{T}=\frac{H_{c}\alpha_{2}E^{\prime }}{{\left(\sqrt{\alpha_{2}E^{\prime }}-\sqrt{\alpha_{1}H_{c}}\right)}^{2}}\;\;\;\;$$

where *H_c_* is contact hardness and Poisson’s ratio of an Al alloy can be taken as 0.284 [41]. The calculated values of resistance to plasticity are given in Table 1.

**Figure 11:**
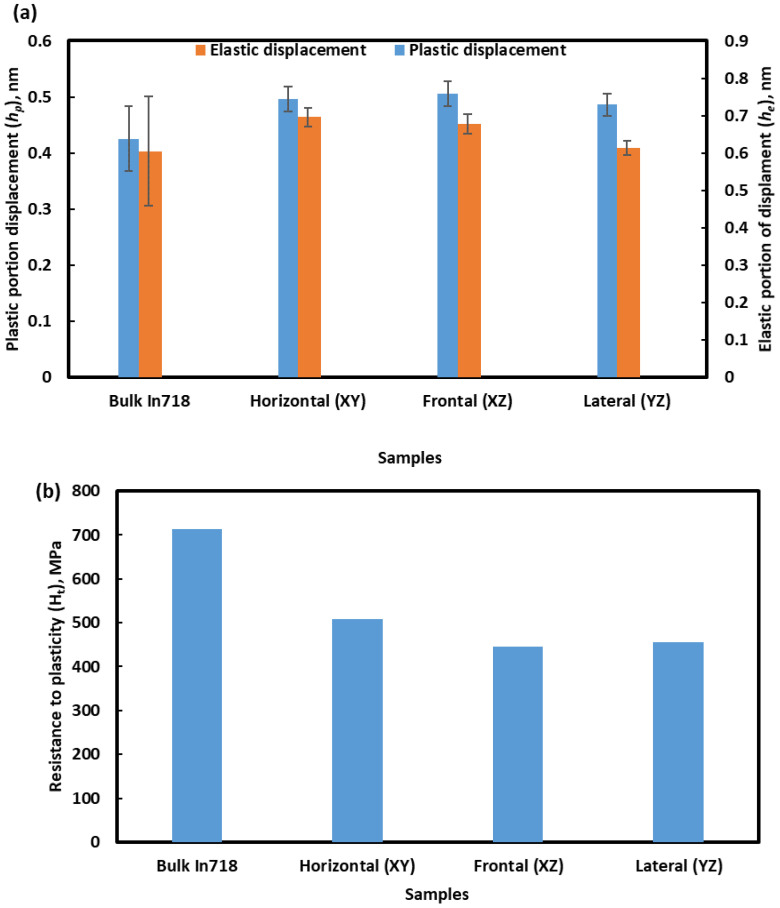
(**a**) Elastic (*h_e_*) and plastic (*h_p_*) displacements and (**b**) resistance to plasticity (*Ht*) of presently investigated Inconel 718 alloys.

Figure 11a shows the elastic and plastic portion of the displacement, which was recorded during nanoindentation. Unsurprisingly, both the elastic and plastic displacements in the cast alloy were lower than those of the L-PBF-processed alloy. Again, in the case of L-PBF-processed alloy, elastic and plastic displacement on horizontal plane are marginally smaller than those of frontal and lateral planes. Figure 11b exhibits the resistance to plasticity (*H_T_*), and it is about 1.4–1.6 times higher for cast alloy than for L-PBF alloy products.

In addition to the resistance to plasticity, maximum shear stresses of the alloys can be calculated by the following relation [42]:

(5) τmax=0.45 (16PcEr29π3R2)13

where *P_c_* denotes the critical load to initiate the plasticity, *E_r_* represents the reduced modulus and *R* is the indenter radius. In the present case, Berkovich indenter tip diameter was about 150 nm (as determined from SEM image). In Equation (4), the critical load *P_c_* is the load when the first pop-in takes place in the loading curve during nanoindentation. However, the loading curves were smooth (Figure 8) in the present case without any indication of cut-offs. In addition, according to the Hertzian contact theory, load *P* can be described as follows [43]:

(6) $$P=\frac{4}{3}E_{r}R^{\frac{1}{2}}h^{\frac{3}{2}}$$

where *h* is the instantaneous indentation depth. Thus, the critical load *P_c_* can be expressed as:

(7) $$P_{c}=\frac{4}{3}E_{r}R^{\frac{1}{2}}h_{e}{}^{\frac{3}{2}}$$

The maximum shear stress of the presently investigated alloys calculated according to Equation (4) and given in Table 1. The maximum shear stress of the cast alloy was about 986 MPa, whereas it was 884–956 MPa for the L-PBF-processed alloy. Here, again, the mechanical properties of the L-PBF alloy were outperformed by others allots. This was withstanding the fact that the grain size of the cast alloy was about five times higher than that of L-PBF alloy. As stated before, this must be due to the absence of precipitate/solid solution strengthening mechanism in the L-PBF alloy [33], as it was not solution/heat-treated.

## 4. Conclusions

The microstructural and nano-mechanical properties of the L-PBF-processed Inconel 718 alloy were investigated in the current study and compared with the cast alloy of similar composition. Based on the presented results and discussion, the following conclusions could be made:

➢ The microstructure of L-PBF-processed alloy is totally different and unique, being composed of cellular structure, melt-pool boundaries and grain boundaries. EBSD analysis confirms the larger grain size (~10 µm) of the cast alloy compared to the smaller cell size (~100–600 nm) of the L-PBF alloy.
➢ The hardness of the L-PBF-processed alloy (279–349 MPa) was lower than that of cast alloy (408 MPa). The resistance of plasticity of the L-PBF-processed alloy was about 1.4–1.6 times lower than the cast alloy, which also gave rise to higher shear stresses of the cast alloy. The maximum shear stress of the L-PBF process alloy was in the range of 884–956 MPa, in comparison to 986 MPa for the cast alloy.
➢ The somewhat inferior mechanical properties of the L-PBF alloy, compared to those of the cast alloy, is induced by the absence of any kind of heat/solution treatment of the L-PFB alloy that favors the solid solution strengthening/precipitation hardening. Therefore, it is recommended that researchers carry out appropriate heat treatment/aging of such alloys prior to any application.

## Figures and Tables

**Table 1:** Physical and mechanical properties of the currently investigated Inconel 718 alloy fabricated by L-PBF and casting process.

Properties	P-LBF-Processed Inconel 718 Alloy			Cast Inconel 718 Alloy
	Lateral (YZ) Plane	Frontal (XZ) Plane	Horizontal (XY) Plane	
Density (gm/cc)	8.08			8.2
Hardness (HV_0.3_)	330 ± 8.05	279.45 ± 9.45	349.18 ± 10.73	408.82 ± 1303
Hardness (MPa)	337.52 ± 23.71	331.32 ± 17.30	372.5 ± 21.03	502.66 ± 21.95
Young’s modulus (GPa)	88.65 ± 3.81	90.11 ± 2.43	92.78 ± 5.77	99.67 ± 6.94
Resistance to plasticity (MPa)	530.19	531.62	529.69	288.83
Maximum shear stresses (GPa)	884.25	945.20	956.25	986.64

## Data Availability

The raw/processed data used to produce the results will be made available by the corresponding author upon reasonable request.

## Supplementary Materials

The following supporting information can be downloaded at: https://www.mdpi.com/article/10.3390/ma16062383/s1, Figure S1: Representative load-displacement graphs on the frontal planes of L-PBF processed Inconel 718 alloy with respect to the build direction (BD); Figure S2: Representative load-displacement graphs on the lateral planes of L-PBF processed Inconel 718 alloy with respect to the build direction (BD); Figure S3: Representative load-displacement graphs on the horizontal planes of the L-PBF processed Inconel 718 alloy with respect to the build direction (BD); Figure S4: Representative load-displacement graphs on the cast Al-12Si alloy.
