# Supplementary material for: Investigation into the Microstructure and Hardness of Additively Manufactured (3D-Printed) Inconel 718 Alloy
Source: Materials (Basel). 2023 Mar 16;16(6):2383. doi: 10.3390/ma16062383 (PMC10051848; doi:10.3390/ma16062383)
Supplement: Supplementary file 1 [file materials-16-02383-s001.zip › materials-2261217-supplementary.pdf]

Supplementary materials

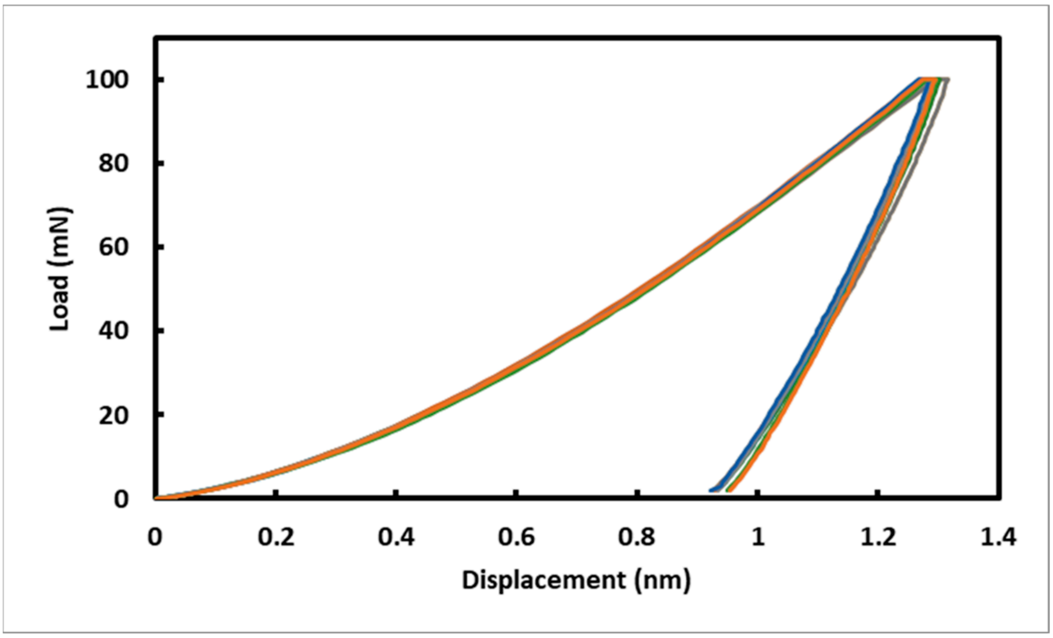

Fig. S1: Representative load-displacement graphs on the frontal planes of L-PBF processed Inconel 718 alloy with respect to the build direction (BD).

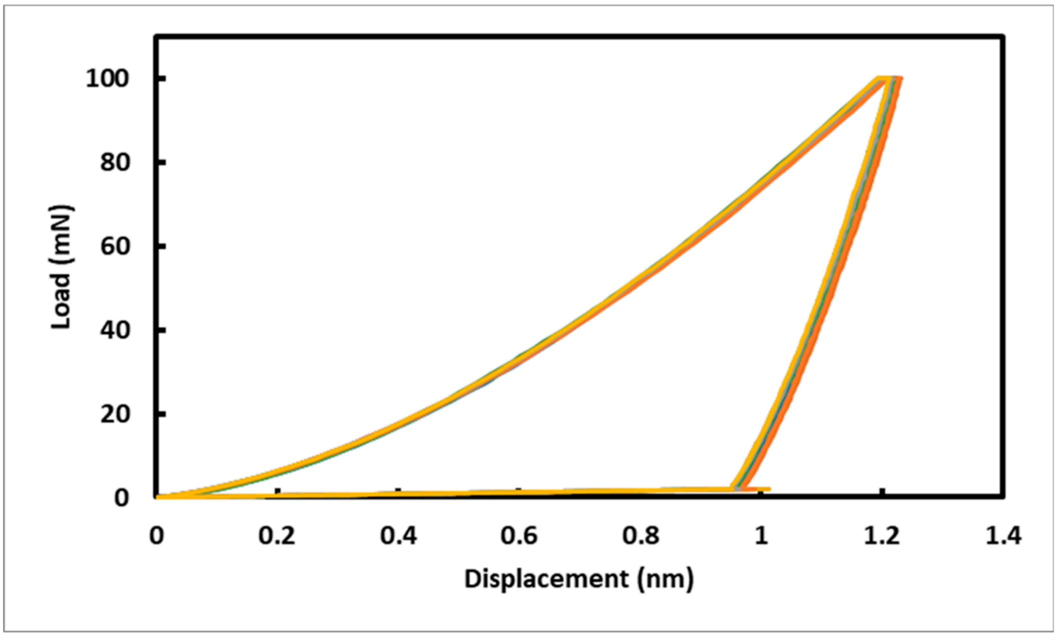

Fig. S2: Representative load-displacement graphs on the lateral planes of L-PBF processed Inconel 718 alloy with respect to the build direction (BD).

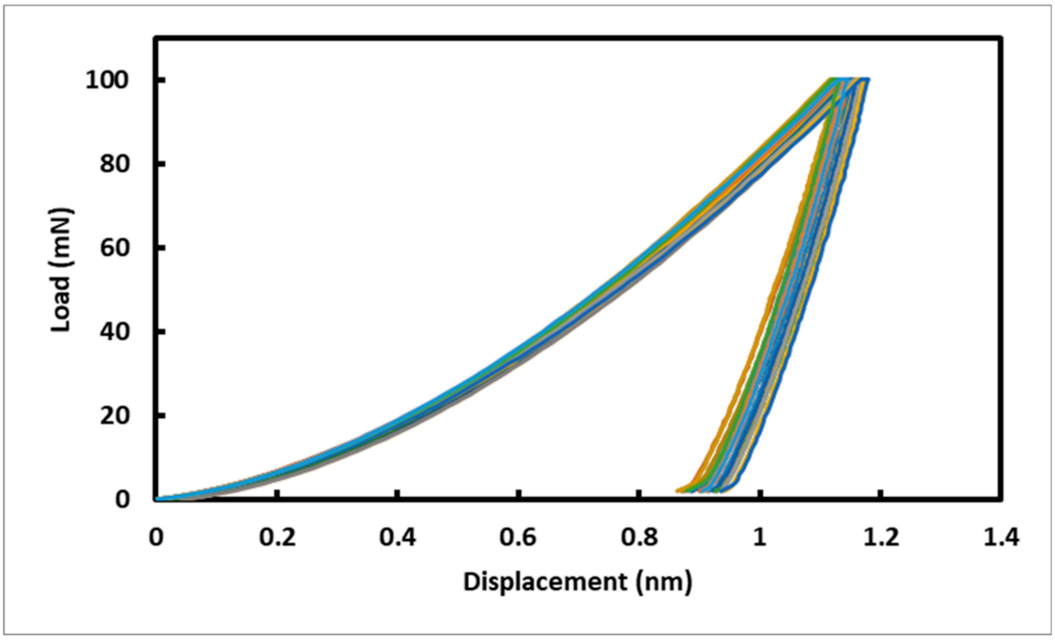

Fig. S3: Representative load-displacement graphs on the horizontal planes of the L-PBF processed Inconel 718 alloy with respect to the build direction (BD).

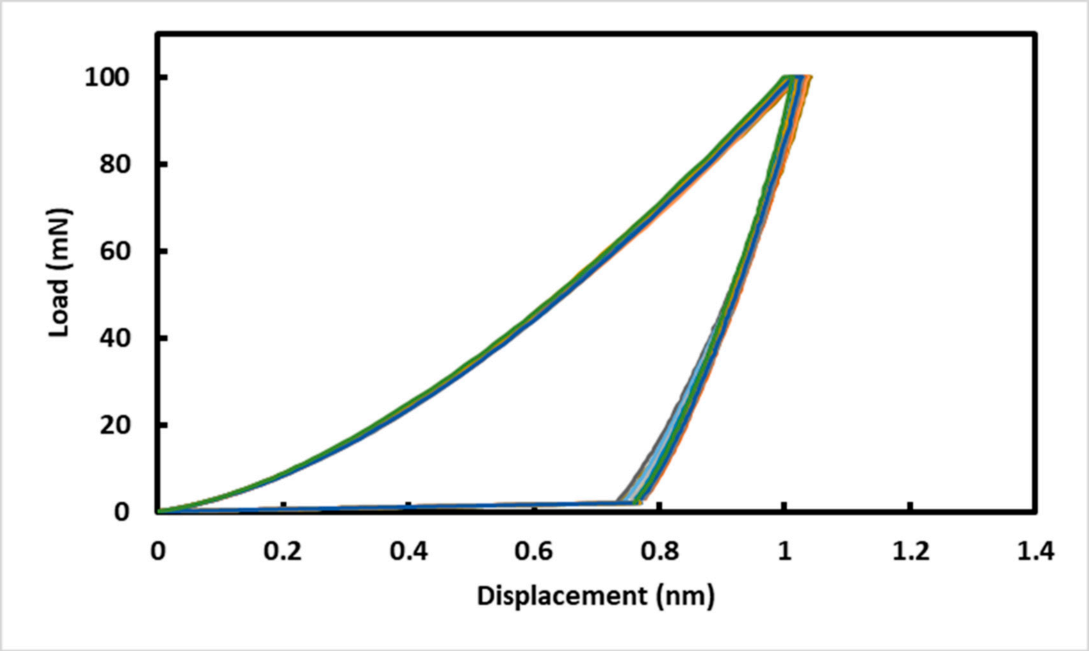

Fig. S4: Representative load-displacement graphs on the cast Al-12Si alloy.
